# Supplementary material for: Deletion of wfs1 Impairs Oligodendrocyte Precursor Cells Dorsal Distribution and Myelination Through the wfs1-hmgcs1 Axis in Zebrafish
Source: Biology (Basel). 2026 Aug 16;15(16):1402. doi: 10.3390/biology15161402 (PMC13510151; doi:10.3390/biology15161402)
Supplement: Supplementary file 1 [file biology-15-01402-s001.zip › File S1-Transcriptome sequencing data and differentially expressed genes (DEGs) in wfs1-- zebrafish at 5 dpf/2.KEGG/wfs1KOvsWT/GSEA/wfs1KO/NUCLEOTIDE_METABOLISM/NUCLEOTIDE_METABOLISM(DRE01232).html]

Details for gene set NUCLEOTIDE\_METABOLISM(DRE01232)[GSEA]

|  || Dataset | deg.sample |
| Phenotype | sample.cls |
| Upregulated in class | wfs1KO |
| GeneSet | NUCLEOTIDE\_METABOLISM(DRE01232) |
| Enrichment Score (ES) | 0.3099296 |
| Normalized Enrichment Score (NES) | 1.3859621 |
| Nominal p-value | 0.025896415 |
| FDR q-value | 0.23247832 |
| FWER p-Value | 0.906 |
Table: GSEA Results Summary

  

Fig 1: Enrichment plot: NUCLEOTIDE\_METABOLISM(DRE01232)      
 Profile of the Running ES Score & Positions of GeneSet Members on the Rank Ordered List

  

| PROBE | DESCRIPTION (from dataset) | GENE SYMBOL | GENE\_TITLE | RANK IN GENE LIST | RANK METRIC SCORE | RUNNING ES | CORE ENRICHMENT || 1 | 100330864 | LOC100330864 |  |  | 70 | 4.818 | 0.0529 | Yes |
| 2 | 492517 | zgc:103586 |  |  | 528 | 2.910 | 0.0719 | Yes |
| 3 | 553639\_1 | gmpr |  |  | 557 | 2.830 | 0.1034 | Yes |
| 4 | 103910413\_1 | tymp |  |  | 1012 | 2.083 | 0.1129 | Yes |
| 5 | 334431\_1 | adsl |  |  | 1025 | 2.072 | 0.1362 | Yes |
| 6 | 558466\_1 | uckl1b |  |  | 1053 | 2.045 | 0.1588 | Yes |
| 7 | 550560\_1 | entpd6 |  |  | 1083 | 2.011 | 0.1809 | Yes |
| 8 | 100005763 | uck2a |  |  | 1117 | 1.979 | 0.2025 | Yes |
| 9 | 402953\_1 | pnp6 |  |  | 1279 | 1.829 | 0.2184 | Yes |
| 10 | 333997\_1 | ampd3b |  |  | 1407 | 1.721 | 0.2341 | Yes |
| 11 | 393744\_1 | dctpp1 |  |  | 1425 | 1.706 | 0.2531 | Yes |
| 12 | 393113 | upp2 |  |  | 1678 | 1.557 | 0.2629 | Yes |
| 13 | 560486 | xdh |  |  | 1812 | 1.458 | 0.2754 | Yes |
| 14 | 324134\_1 | asmtl |  |  | 1924 | 1.399 | 0.2880 | Yes |
| 15 | 556849 | entpd5a |  |  | 2149 | 1.307 | 0.2959 | Yes |
| 16 | 447905 | entpd2a.1 |  |  | 2728 | 1.102 | 0.2903 | Yes |
| 17 | 333997 | ampd3b |  |  | 2884 | 1.062 | 0.2975 | Yes |
| 18 | 791647 | pnp5a |  |  | 2948 | 1.051 | 0.3076 | Yes |
| 19 | 562627\_1 | nt5c1aa |  |  | 3317 | 0.969 | 0.3071 | Yes |
| 20 | 327590 | tk1 |  |  | 3561 | 0.918 | 0.3099 | Yes |
| 21 | 570547 | uckl1a |  |  | 4313 | 0.774 | 0.2951 | No |
| 22 | 436692 | entpd4 |  |  | 4461 | 0.750 | 0.2991 | No |
| 23 | 393559 | gmps |  |  | 4958 | 0.686 | 0.2913 | No |
| 24 | 445201 | cant1b |  |  | 5037 | 0.677 | 0.2966 | No |
| 25 | 393906 | nt5e |  |  | 5288 | 0.648 | 0.2962 | No |
| 26 | 437016 | tk2 |  |  | 6454 | 0.541 | 0.2657 | No |
| 27 | 368909 | rrm2b |  |  | 6644 | 0.524 | 0.2657 | No |
| 28 | 100005551 | entpd3 |  |  | 6753 | 0.515 | 0.2682 | No |
| 29 | 556578 | ampd3a |  |  | 6885 | 0.501 | 0.2698 | No |
| 30 | 570478 | cmpk2 |  |  | 6893 | 0.501 | 0.2753 | No |
| 31 | 30086 | nme7 |  |  | 7162 | 0.476 | 0.2723 | No |
| 32 | 553671\_1 | ak8 |  |  | 7165 | 0.476 | 0.2777 | No |
| 33 | 100005054 | lacc1 |  |  | 7184 | 0.475 | 0.2826 | No |
| 34 | 553701 | gda |  |  | 7572 | 0.450 | 0.2756 | No |
| 35 | 567734 | dtymk |  |  | 7842 | 0.436 | 0.2721 | No |
| 36 | 550332 | dctd |  |  | 8065 | 0.418 | 0.2699 | No |
| 37 | 445245 | ctps1b |  |  | 8427 | 0.408 | 0.2632 | No |
| 38 | 445151 | entpd1 |  |  | 8827 | 0.379 | 0.2550 | No |
| 39 | 100538266 | LOC100538266 |  |  | 8883 | 0.375 | 0.2575 | No |
| 40 | 402940 | pnp4b |  |  | 8950 | 0.371 | 0.2597 | No |
| 41 | 503707 | upp1 |  |  | 9292 | 0.347 | 0.2529 | No |
| 42 | 445069 | ntpcr |  |  | 9362 | 0.343 | 0.2547 | No |
| 43 | 406558 | cant1a |  |  | 9674 | 0.323 | 0.2486 | No |
| 44 | 431724 | impdh1a |  |  | 9905 | 0.310 | 0.2449 | No |
| 45 | 436919 | ada |  |  | 10721 | 0.259 | 0.2222 | No |
| 46 | 100003595 | ak5 |  |  | 10724 | 0.259 | 0.2251 | No |
| 47 | 110438480 | LOC110438480 |  |  | 10875 | 0.250 | 0.2232 | No |
| 48 | 474325 | dck |  |  | 11056 | 0.241 | 0.2203 | No |
| 49 | 58120 | nme6 |  |  | 11325 | 0.226 | 0.2145 | No |
| 50 | 406259 | hprt1 |  |  | 11719 | 0.205 | 0.2044 | No |
| 51 | 559295 | cecr1b |  |  | 11760 | 0.203 | 0.2055 | No |
| 52 | 30086\_1 | nme7 |  |  | 11771 | 0.202 | 0.2075 | No |
| 53 | 406404 | ak3 |  |  | 11960 | 0.193 | 0.2038 | No |
| 54 | 406590 | ak4 |  |  | 11966 | 0.193 | 0.2058 | No |
| 55 | 541538 | zgc:110540 |  |  | 12538 | 0.162 | 0.1897 | No |
| 56 | 571355 | ampd2b |  |  | 12561 | 0.162 | 0.1909 | No |
| 57 | 557834 | itpa |  |  | 12663 | 0.156 | 0.1895 | No |
| 58 | 415216 | guk1a |  |  | 12813 | 0.149 | 0.1865 | No |
| 59 | 447889 | pnp5b |  |  | 13386 | 0.121 | 0.1699 | No |
| 60 | 30740 | rrm1 |  |  | 13918 | 0.096 | 0.1543 | No |
| 61 | 192305 | adssl |  |  | 14103 | 0.087 | 0.1495 | No |
| 62 | 81540 | tyms |  |  | 14768 | 0.057 | 0.1292 | No |
| 63 | 557972 | nt5c2b |  |  | 15006 | 0.046 | 0.1223 | No |
| 64 | 324845 | nt5c2a |  |  | 15095 | 0.041 | 0.1200 | No |
| 65 | 393697 | guk1b |  |  | 15337 | 0.031 | 0.1127 | No |
| 66 | 447928 | uck1 |  |  | 15524 | 0.021 | 0.1071 | No |
| 67 | 436652 | entpd8 |  |  | 15584 | 0.018 | 0.1055 | No |
| 68 | 394170 | nme4 |  |  | 15678 | 0.013 | 0.1027 | No |
| 69 | 562399 | enpp1 |  |  | 17165 | -0.039 | 0.0563 | No |
| 70 | 431724\_2 | impdh1a |  |  | 17661 | -0.061 | 0.0415 | No |
| 71 | 449835 | dut |  |  | 18046 | -0.078 | 0.0303 | No |
| 72 | 563597 | si:ch211-243j20.2 |  |  | 18339 | -0.091 | 0.0221 | No |
| 73 | 560627 | LOC560627 |  |  | 18375 | -0.093 | 0.0221 | No |
| 74 | 415208 | zgc:86811 |  |  | 18397 | -0.093 | 0.0225 | No |
| 75 | 406383 | cmpk |  |  | 18494 | -0.099 | 0.0206 | No |
| 76 | 368220 | adka |  |  | 18659 | -0.106 | 0.0166 | No |
| 77 | 553671\_2 | ak8 |  |  | 18745 | -0.111 | 0.0152 | No |
| 78 | 100005067\_2 | dguok |  |  | 18799 | -0.113 | 0.0149 | No |
| 79 | 407989 | adssl1 |  |  | 18908 | -0.118 | 0.0128 | No |
| 80 | 678545 | gmpr2 |  |  | 18925 | -0.119 | 0.0137 | No |
| 81 | 402979 | cda |  |  | 19179 | -0.130 | 0.0072 | No |
| 82 | 561683 | nt5c1bb |  |  | 19180 | -0.130 | 0.0087 | No |
| 83 | 504040 | ak7b |  |  | 19290 | -0.135 | 0.0068 | No |
| 84 | 393867 | ampd1 |  |  | 19427 | -0.140 | 0.0041 | No |
| 85 | 321793 | ak2 |  |  | 20598 | -0.193 | -0.0305 | No |
| 86 | 415192 | pnp4a |  |  | 20628 | -0.195 | -0.0292 | No |
| 87 | 445486 | ak1 |  |  | 20697 | -0.198 | -0.0291 | No |
| 88 | 30084 | nme2b.2 |  |  | 20707 | -0.199 | -0.0271 | No |
| 89 | 557708 | si:ch211-199m3.4 |  |  | 20731 | -0.200 | -0.0255 | No |
| 90 | 393641 | aprt |  |  | 21266 | -0.228 | -0.0397 | No |
| 91 | 553639 | gmpr |  |  | 21373 | -0.235 | -0.0404 | No |
| 92 | 559298 | entpd2a.2 |  |  | 21728 | -0.253 | -0.0486 | No |
| 93 | 100536156 | LOC100536156 |  |  | 21946 | -0.264 | -0.0524 | No |
| 94 | 317745 | impdh2 |  |  | 22289 | -0.283 | -0.0599 | No |
| 95 | 562627 | nt5c1aa |  |  | 22497 | -0.296 | -0.0631 | No |
| 96 | 449987 | zgc:103759 |  |  | 22590 | -0.301 | -0.0625 | No |
| 97 | 447810 | nt5c2l1 |  |  | 22960 | -0.324 | -0.0704 | No |
| 98 | 322089 | ctps1a |  |  | 23055 | -0.331 | -0.0696 | No |
| 99 | 321720 | nt5c3a |  |  | 23073 | -0.332 | -0.0663 | No |
| 100 | 30083 | nme2b.1 |  |  | 23078 | -0.333 | -0.0627 | No |
| 101 | 322229 | adkb |  |  | 23529 | -0.363 | -0.0727 | No |
| 102 | 550502 | adss |  |  | 23578 | -0.365 | -0.0700 | No |
| 103 | 402953 | pnp6 |  |  | 23940 | -0.388 | -0.0769 | No |
| 104 | 570547\_1 | uckl1a |  |  | 24045 | -0.396 | -0.0757 | No |
| 105 | 30085 | nme3 |  |  | 24681 | -0.428 | -0.0908 | No |
| 106 | 406259\_1 | hprt1 |  |  | 24744 | -0.434 | -0.0878 | No |
| 107 | 334431\_2 | adsl |  |  | 25123 | -0.455 | -0.0945 | No |
| 108 | 799449 | nt5c1ab |  |  | 25372 | -0.473 | -0.0969 | No |
| 109 | 559958 | cdaa |  |  | 25499 | -0.485 | -0.0953 | No |
| 110 | 445245\_1 | ctps1b |  |  | 25666 | -0.500 | -0.0948 | No |
| 111 | 335716 | nme2a |  |  | 26542 | -0.583 | -0.1157 | No |
| 112 | 338306 | impdh1b |  |  | 26651 | -0.593 | -0.1123 | No |
| 113 | 798022 | nt5c1ba |  |  | 26775 | -0.606 | -0.1092 | No |
| 114 | 393744 | dctpp1 |  |  | 27009 | -0.629 | -0.1093 | No |
| 115 | 327057 | uck2b |  |  | 27131 | -0.641 | -0.1058 | No |
| 116 | 554129 | hddc2 |  |  | 27728 | -0.706 | -0.1165 | No |
| 117 | 550560 | entpd6 |  |  | 28271 | -0.781 | -0.1246 | No |
| 118 | 373884 | cecr1a |  |  | 28385 | -0.801 | -0.1190 | No |
| 119 | 415146 | hprt1l |  |  | 28695 | -0.857 | -0.1189 | No |
| 120 | 100330772 | ampd2a |  |  | 28873 | -0.894 | -0.1143 | No |
| 121 | 569142 | entpd2b |  |  | 29566 | -1.083 | -0.1237 | No |
| 122 | 100005067\_1 | dguok |  |  | 29683 | -1.122 | -0.1145 | No |
| 123 | 324134 | asmtl |  |  | 29875 | -1.193 | -0.1068 | No |
| 124 | 30733 | rrm2 |  |  | 29935 | -1.219 | -0.0948 | No |
| 125 | 393744\_2 | dctpp1 |  |  | 30099 | -1.285 | -0.0852 | No |
| 126 | 558466 | uckl1b |  |  | 30217 | -1.346 | -0.0735 | No |
| 127 | 103910413 | tymp |  |  | 30218 | -1.348 | -0.0580 | No |
| 128 | 548336 | ak5l |  |  | 30260 | -1.372 | -0.0436 | No |
| 129 | 437016\_1 | tk2 |  |  | 30898 | -1.862 | -0.0424 | No |
| 130 | 100005763\_1 | uck2a |  |  | 30953 | -1.908 | -0.0222 | No |
| 131 | 492517\_1 | zgc:103586 |  |  | 31787 | -4.517 | 0.0032 | No |
Table: GSEA details [plain text format]

  

Fig 2: NUCLEOTIDE\_METABOLISM(DRE01232)      
 Blue-Pink O' Gram in the Space of the Analyzed GeneSet

  

Fig 3: NUCLEOTIDE\_METABOLISM(DRE01232): Random ES distribution      
 Gene set null distribution of ES for **NUCLEOTIDE\_METABOLISM(DRE01232)**
